# A Targeted *RNAi* Screen Reveals *Drosophila* Female-Sterile Genes That Control the Size of Germline Stem Cell Niche During Development

**DOI:** 10.1534/g3.118.200355

**Published:** 2018-05-15

**Authors:** Yueh Cho, Chun-Ming Lai, Kun-Yang Lin, Hwei-Jan Hsu

**Affiliations:** * Institute of Cellular and Organismic Biology, Academia Sinica; † Molecular and Biological Agricultural Sciences Program, Taiwan International Graduate Program, National Chung Hsing University and Academia Sinica, Taipei 11529, Taiwan

**Keywords:** GSC, stem cell, stem cell niche, insulin

## Abstract

Adult stem cells maintain tissue homeostasis. This unique capability largely depends on the stem cell niche, a specialized microenvironment, which preserves stem cell identity through physical contacts and secreted factors. In many cancers, latent tumor cell niches are thought to house stem cells and aid tumor initiation. However, in developing tissue and cancer it is unclear how the niche is established. The well-characterized germline stem cells (GSCs) and niches in the *Drosophila melanogaster* ovary provide an excellent model to address this fundamental issue. As such, we conducted a small-scale *RNAi* screen of 560 individually expressed *UAS-RNAi* lines with targets implicated in female fertility. *RNAi* was expressed in the soma of larval gonads, and screening for reduced egg production and abnormal ovarian morphology was performed in adults. Twenty candidates that affect ovarian development were identified and subsequently knocked down in the soma only during niche formation. Feminization factors (Transformer, Sex lethal, and Virilizer), a histone methyltransferase (Enhancer of Zeste), a transcriptional machinery component (Enhancer of yellow 1), a chromatin remodeling complex member (Enhancer of yellow 3) and a chromosome passenger complex constituent (Incenp) were identified as potentially functioning in the control of niche size. The identification of these molecules highlights specific molecular events that are critical for niche formation and will provide a basis for future studies to fully understand the mechanisms of GSC recruitment and maintenance.

The stem cell niche functions to recruit stem cells during tissue development and maintain these cells throughout the life of the organism. Therefore, establishment of the niche is a critical aspect of all stem cell systems. However, little is known about the mechanisms that govern this process.

To address the fundamental question of how stem cell niches are established, the *Drosophila* ovary can be considered an excellent model, based on its well-characterized cell biology. One *Drosophila* female has a single pair of ovaries (Figure 1A). Each ovary is composed 16-20 ovarioles, which are the functional units that produce eggs (Spradling 1993). The anterior-most structure of the ovariole is the germarium (Figure 1B); the anterior tip of the germarium is constructed from a terminal filament (TF), 4-6 cap cells and anterior escort cells, which together create a germline stem cell (GSC) niche that houses two to three GSCs (Wong *et al.*, 2005). GSCs form direct contacts with cap cells, and each GSC contains one fusome, which is juxtaposed to the interface between the GSC and the cap cell (de Cuevas and Spradling 1998). Cap cells are considered to be the major component of the GSC niche due to their production of BMP stemness factors and E-cadherin-mediated physical contact with GSCs (Song *et al.*, 2002; Xie and Spradling 2000). Each progeny that is destined for differentiation from an asymmetric GSC division is called a cystoblast and undergoes four incomplete divisions to become a 16-cell cyst; within the cyst, the germ cells are interconnected by a branched fusome.

**Figure 1:**
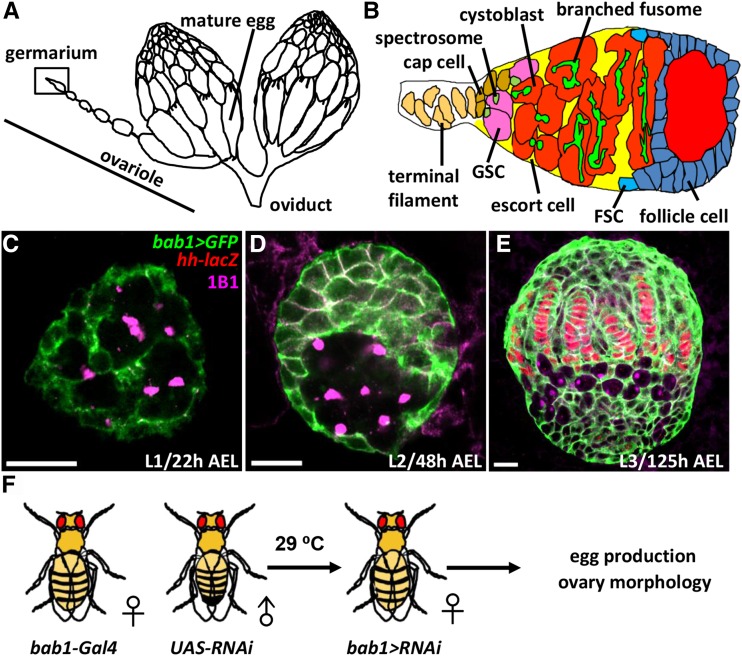
The *Drosophila* ovary and germarium, *bab1-GAL4* expression in larval ovaries, and the screening strategy. (A) A female fly has two ovaries that are bridged by an oviduct. Each ovary is composed of 16-20 ovarioles, which produce mature eggs. The anterior-most structure is called the germarium. (B) In the germarium, terminal filament (TF) and cap cells form the GSC niche, which houses two to three GSCs that directly interact with cap cells. Each GSC carries a cytoplasmic organelle, called a fusome (spectrosome). GSC progeny undergo four rounds of incomplete division to form 16-cell cysts; each cell in the cyst is interconnected to the others by a branched fusome. Germ cells are first wrapped by escort cells and then by follicle cells, which are derived from follicle stem cells (FSCs), to form egg chambers. (C-E) L1 (A), L2 (B) and L3-stage larval ovaries with *bab1 > gfp* (green), *hh-lacZ* (red, developing terminal filament (TF) and cap cells), and 1B1 (magenta, fusomes). AEL, after egg laying; h, hours. (D) Scheme for identifying factors that are involved in niche formation. Female virgins carrying *bab1-GAL4* were crossed with male flies carrying *UAS-RNAi*. Their eggs were maintained and cultured until eclosed; female progeny carrying both *bab1-GAL4* and *UAS-RNAi* were collected for an egg laying assay and examination of ovary morphology.

When a *Drosophila* hatches, the larval ovary is a sphere that only contains a small number of primordial germ cells (PGCs), each containing a round-shape fusome called a spectrosome and somatic gonadal precursors (SGPs) (Figure 1C) (Lai *et al.*, 2017). During larval stages (Figure 1C-E), PGCs and SGPs increase in number, and SGPs differentiate into different types of gonadal somatic cells. The first morphogenetic movement along the anterior-posterior and medial-lateral axis of the ovary creates a two-dimensional array of 16-20 stacks of somatic cells called TFs (Sahut-Barnola *et al.*, 1995). TF cells start to form at the late-second instar larval stage (Lai *et al.*, 2017), and gradually increase in number to 8 or 9, displaying a disc-like shape until early pupal stages (Sahut-Barnola *et al.*, 1995). After TF formation, cap cells start to form at the late-third instar larval stage and complete their development in the pupal stage (Zhu and Xie 2003). The final number of cap cells is approximately 4-6 until aging causes a decline (Hsu and Drummond-Barbosa 2009); these cap cells can be distinguished from TF cells because they are rounder and do not align with the TF (Zhu and Xie 2003). PGCs are intermingled with another type of somatic cells, intermingled cells (ICs), and are located in the central region of the larval ovary. During pupation, apical somatic cells migrate basally between TFs to divide the ovary into ovarioles (Cohen *et al.*, 2002).

Despite detailed knowledge of how the GSC niche and the rest of the ovariole are constructed during development, little is known about the molecular mechanisms that control these cellular processes. Among the few studies that have been published on this topic, one concluded that during the larval-pupal transition, Notch signaling is activated in the anterior ICs, directing them to become niche cap cells (Song *et al.*, 2007). Moreover, we previously showed that Hh signaling specifies IC cell fate, distinct from other somatic cells, by controlling cell affinity. Therefore, reducing Hh signaling in ICs resulted in the loss of cap cells (Lai *et al.*, 2017). Mainly owing to a lack of specific molecular markers for cap cells and difficulties in dissecting pupal ovaries – including the small size of larval ovaries and adhesion of degenerated fat cells to the pupal ovary (Park *et al.*, 2018) – the mechanisms that regulate niche cap cell formation remain poorly understood.

In this study, we used the powerful *UAS-GAL4* system to individually drive *RNAi* expression and knockdown genes, which are all known to be involved in female fertility, in the ovarian soma during development. *RNAi* candidates that affected egg production and ovarian morphology were further analyzed for effects on niche formation by evaluating GSC and niche cap cell numbers in the adult germarium. At this stage, the ovaries are easily dissected, and completely formed cap cells can be unambiguously identified by morphology and location. With this screen, we identified seven genes with various functions, including feminization, transcription initiation, and chromatin methylation, remodeling and exchange, which are required for niche cell formation.

## Materials And Methods

### Drosophila Stocks and Culture Conditions

*Drosophila* stocks were maintained on standard sugar/yeast/cornmeal/agar food at 25°, unless indicated. *w^1118^* was used as the control. *UAS-RNAi* lines were obtained from the National Institute of Genetics (NIG-Fly, Japan) or from the Vienna Drosophila Resource Center (VDRC). *bab1-GAL4*, a niche driver (Bolívar *et al.* 2006), was used to drive *RNAi* expression in the gonadal soma during developmental stages. Flies expressing *RNAi* driven by *bab1-GAL4* also carried *tub-GAL80^ts^* to control GAL4 expression (McGuire *et al.*, 2004). At 18°, GAL80ts suppresses the activity of GAL4, which is driven by a specific promoter. At 29°, GAL80^ts^ is degraded, and GAL4 activates expression of *RNAi*. For whole-stage knockdown, flies carrying *bab1-GAL4* and *tub-GAL80^ts^* were individually mated with *UAS-RNAi* lines at 29°; flies were transferred to a new vial every two days and newly eclosed females were collected within one day for ovary dissection. For knockdown from the L3 to the adult stage, crosses were set up at 18° and transferred to a new vial every two days; vials with larvae climbing up and down from the food (signifying third-instar larvae) were switched to 29° and the first batch of eclosed females were collected within one day for ovary dissection.

Other genetic elements are described in Flybase (https://flybase.org).

### Egg Count Measurement and Ovary Imaging

Newly eclosed females were cultured with *w^1118^* males for 2 days at 29°, and then transferred into plastic bottles containing molasses plates with a layer of wet yeast (changed daily). To measure egg production, five pairs of flies per bottle were cultured and the number of eggs laid was counted every 24 h in triplicate. Ovaries from newly enclosed flies or from flies used for the egg counts were dissected at day 5 and imaged (ZEISS AxioCam ERc5s).

### Immunostaining and Fluorescence Microscopy

Ovaries were dissected in Grace’s Insect Medium (Lonza), then fixed for 13 min at room temperature in 5% (vol/vol) paraformaldehyde (Alfa Aesar)/Grace’s Insect Medium, after which the tissues were washed and stained as previously described (Hsu *et al.*, 2008). The following primary antibodies were used: mouse monoclonal 1B1 [1:50; Developmental Studies Hybridoma Bank (DSHB)], mouse monoclonal anti-Lamin (Lam) C (1:50; DSHB), rabbit polyclonal anti-Vasa (1:500; Santa Cruz), and rabbit polyclonal anti-phospho-Mad (1: 500, Abcam #52903). Alexa 488- or Alexa 568-conjugated goat anti-mouse and anti-rabbit secondary antibodies (1:500; Jackson ImmunoResearch) were used. Samples were incubated in 0.5 μg/mL DAPI (Sigma-Aldrich) for 10 min. Ovaries were mounted in 80% glycerol containing 20.0 µg/mL N-propyl gallate (Sigma) and observed on a Zeiss LSM 700 confocal microscope.

All data were recorded in Excel, and a Student’s *t*-test was used to calculate statistically significant differences; *P < 0.05, **P < 0.01, ***P < 0.001.

### Data availability

Strains are all available from the Drosophila fly stock centers. The authors affirm that all data necessary for confirming the conclusions of the article are present within the article, figures, tables and supplementary information. Supplemental material available at Figshare: https://doi.org/10.25387/g3.6268955.

## Results And Discussion

### Identification of somatic factors involved in GSC niche formation via RNAi-based screening

To identify genes involved in niche formation, we conducted a genetic screen using transgenic *UAS-RNAi* lines from the National Institute of Genetics (NIG) and a somatic driver, *bric-a-brac 1* (*bab1*)*-GAL4*. The *bab1-GAL4* line was constructed by an insertion of *P*-element bearing a GAL4 transcription factor in the promoter region of the *bab1* gene (Bolívar *et al.* 2006), which is essential for the organization of TFs and correct ovary morphology (Godt and Laski 1995; Sahut-Barnola *et al.*, 1995). With this driver, *GAL4* is expressed in all somatic cells (Figure 1C-E), but it is highly restricted to TF and cap cells in adults (Bolívar *et al.* 2006). We selected 560 *UAS-RNAi* lines with targets that have been implicated in female fertility and individually crossed them with the *bab1-GAL4* line. In adult flies, we screened for reduced egg production and abnormal ovarian morphology (Figure 1F), because both of these phenotypes may reflect defects in SGP development, including cap cell precursors, and are readily observable. Crosses were made at 29°, such that *RNAi* would be expressed and knockdown targets throughout development. Newly eclosed flies were collected and cultured with males for an additional two days, after which an egg laying assay was performed. Ovaries were subsequently dissected for morphological observation.

It has been previously reported that the insulin signaling pathway plays a role in controlling *Drosophila* larval ovary size, niche cell number and GSC differentiation (Gancz and Gilboa 2013). Disruption of insulin signaling in the soma results in an extremely small larval ovary, accompanied by reductions in niche cell number and PGC differentiation. To validate our screen, we first knocked down *Drosophila insulin receptor* (*dInR*) using an *RNAi* line driven by *bab1-GAL4*. We found that 2-day-old *dInR*-knockdown (*KD*) ovaries were smaller than those in control flies, with significantly reduced egg production, in addition to diminished GSC and niche cap cell numbers (Figure S1). Based on these results, we conclude that our screen is able to identify somatic factors that affect niche formation.

**Figure 2:**
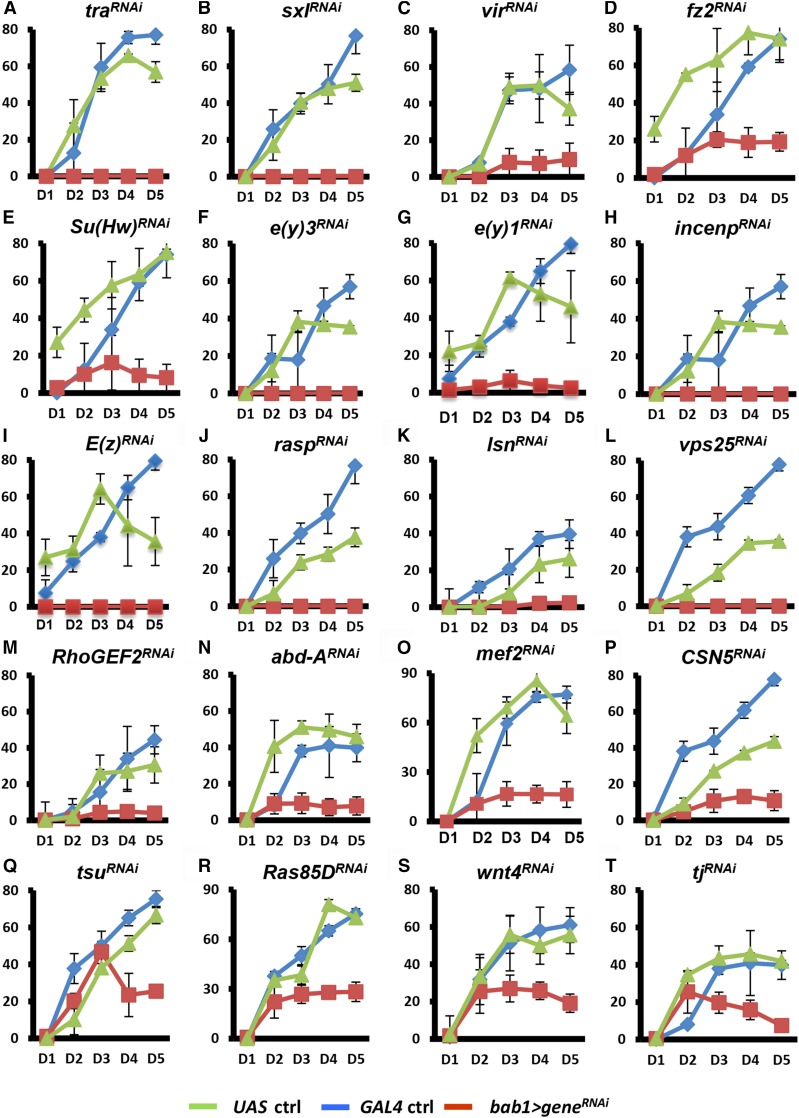
Candidates are necessary for egg production. The egg laying assay was performed on 2-day (D)-old *GAL4* control (ctrl), *UAS* control and *bab1 > gene^RNAi^* females for 5 days. The genotype of the *UAS* control is *UAS-gene^RNAi^/*+ and the *GAL4* control is *bab1-GAL4/*+.

**Figure 3:**
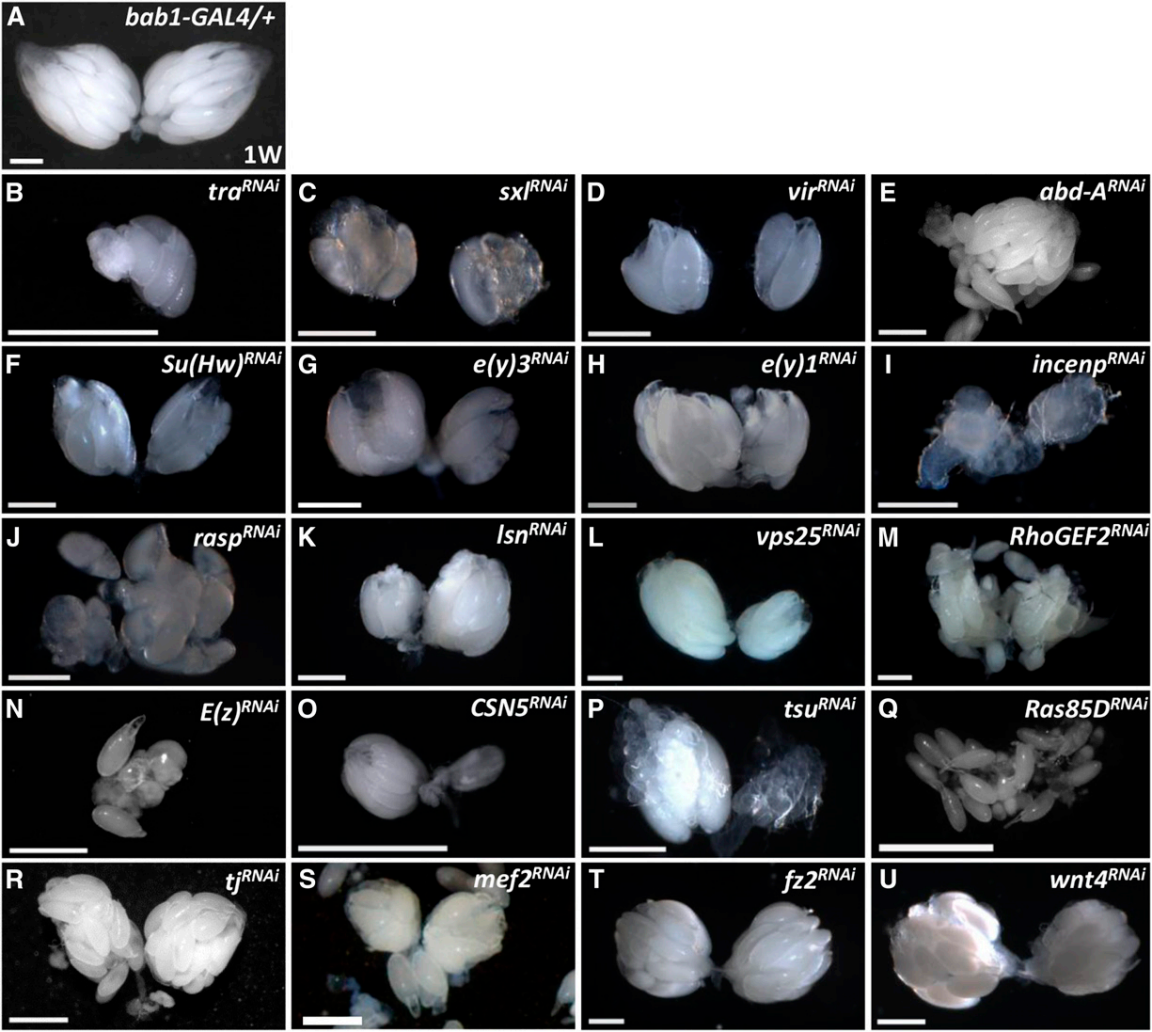
Candidates control ovary morphology. One-week (W)-old *GAL4* control (A) and *bab1 > gene^RNAi^* ovaries (B-U). Scale bar, 50 μm. The genotype of the *GAL4* control is *bab1-GAL4/*+.

Throughout our screen, 20 genes were identified as functioning in the ovarian soma during development to stimulate functional reproduction (Figure 2) and normal ovary morphology (Figure 3). For example, *UAS* (*UAS-transform (tra)^RNAi^ /+*) and *GAL4* control females (*bab1-GAL4/+*) produced approximately 60-80 eggs on the fourth day of the egg laying assay; however, females with *traKD* were completely sterile (Figure 3A). We also dissected the ovaries after the egg laying assay to evaluate morphology. In the control ovary (Figure 3A), the transparent portion was composed of germarium and previtellogenic egg chambers while vitellogenic egg chambers were white from yolk accumulation. In contrast, ovaries of the 20 candidates were smaller (for example, Figure 3B-D), had lost the transparent portion (for example, Figure 3K and L), or exhibited dispersed eggs (Figure 3J, M and Q). Such morphological disruptions clearly indicate that these genes contribute to the normal architecture of ovaries.

We subdivided the candidates into six groups (Table 1), according to their known function. The first subgroup consists of DNA/RNA regulators, including Transformer (Tra), Sex lethal (Sxl), Virilizer (Vir), Tsunagi (Tsu) and Enhancer of zeste [E(z)]. Tra, Sxl and Vir function in RNA-splicing to control female somatic sexual differentiation (Penalva and Sánchez 2003). Vir regulates female-specific splicing of Sxl, and the female isoform of Sxl controls splicing of Tra, which activates *doublesex* female specific splicing by promoting the activity of a splicing enhancer complex (Sciabica and Hertel 2006). Tsu is an RNA-binding protein that forms a complex with Mago Nashi to establish polarity and localize *oskar* mRNA during *Drosophila* oogenesis (Mohr *et al.*, 2001). E(z) is the catalytic component of the Polycomb Repressive Complex 2 (PRC2) methyltransferase that methylates histone H3 lysine 27 (Lund and van Lohuizen 2004), which then recruits PRC1 to silence developmental genes and determine specific differentiated cell identities (Cao and Zhang 2004).

**Table 1:** Candidates identified from the RNAi screen control egg production and ovary morphology

Gene	CG name	Function of encoded protein	Human Ortholog
**DNA/RNA regulators**			
*tra*	CG16724	RNA splicing	—
*sxl*	CG43770	RNA splicing	—
*vir*	CG3496	RNA splicing	*KIAA1429*
*tsu*	CG8781	RNA splicing	*RBM8A*
*E(z)*	CG6502	Histone methylation	*EZH1/EZH2*
**Transcription factors/regulators**			
*abd-A*	CG10325	Homeobox transcription factor	—
*tj*	CG10034	Maf transcription factor	*MAF*
*mef2*	CG1429	Transcription factor	*MEF2D*
*Su(Hw)*	CG8573	Transcription factor and Insulator binding	*ZNF726*
*e(y)3*	CG12238	Component of ATP-dependent chromatin remodeling complexes	*PHF10/BAF45A*
*e(y)1*	CG6474	Component of the Transcription factor II D complex	*TAF9B*
**Transport complex proteins**			
*lsn*	CG6637	Component of endosomal sorting complex	*SNF8*
*vps25*	CG14750	Component of endosomal sorting complex	*VPS25*
**Small GTPase and guanine nucleotide exchange factor**			
*ras85D*	CG9375	Small GTPase	*RAS*
*rhoGEF2*	CG9635	Rho-type guanine nucleotide exchange factor	*PDZ-RHOGEF*
**Wnt signaling components**			
*fz2*	CG9739	Wnt protein binding	*FZD1-10*
*wnt4*	CG4698	Wnt receptor signaling	*WNT4*
**Others**			
*CSN5*	CG14884	Component of COP9 signalosomes	*COPS5*
*incenp*	CG12165	Component of chromosome passenger complex	*INCENP*
*rasp*	CG11495	Acyltransferase enzyme	*-*

The candidates in the second group are transcription factors or regulators. Abdominal A (Abd A) is a homeobox-containing transcription factor and contributes to the developmental fate of embryonic segments (Foronda *et al.*, 2006). Traffic jam (Tj) is a basic leucine zipper Maf transcription factor that regulates multiple processes in gonad morphogenesis (Li *et al.*, 2003), including controlling the interaction between PGCs and the soma. Elimination of Tj in the ovarian soma results in PGC clustering. Moreover, a recent study has also revealed that mutation of Tj may cause the conversion of cap cell fate to TF fate (Panchal *et al.*, 2017), further validating our screen. Myocyte enhancer factor 2 (Mef2) belongs to the MADS-box family and controls muscle development (Black and Olson 1998). Suppressor of Hairy wing [Su(Hw)] is a zinc finger C2H2 transcription factor and a component of the gypsy chromatin insulator that establishes independent domains of transcriptional activity within eukaryotic genomes (Parnell *et al.*, 2006). Enhancer of yellow 3 [E(y)3] is a component of ATP-dependent chromatin remodeling complexes that regulate nucleosome organization (Shidlovskii *et al.*, 2005). Enhancer of yellow 1 [E(y)1] is a principal component of the Transcription factor II D complex that recruits transcriptional machinery to core promoters and organizes specific enhancer-promoter interactions (Soldatov *et al.*, 1999).

The third group is comprised of components of the Endosomal Sorting Complexes Required for Transport (ESCRT)-II complex (Teo *et al.*, 2004), including Larsen (Lsn, also known as Vacuolar protein sorting 22) and Vacuolar protein sorting 25 (Vps25). The ESCRT-II complex sorts certain endocytosed receptors for degradation, while also regulating Notch trafficking, autophagy and *bicoid* mRNA oocyte localization (Irion and St Johnston 2007; Thompson *et al.*, 2005).

The fourth group are Ras oncogene at 85D (Ras85D), a small GTPase, and Rho Guanine Nucleotide Exchange Factors (RhoGEF2). Ras85D is a member of the Ras superfamily of small GTPases and acts in signal transduction cascades to regulate tissue growth and development (Simon *et al.*, 1991). RhoGEF2 is Guanine Nucleotide Exchange Factor for Rho family GTPases (Schmidt and Hall 2002).

The fifth group consists of Wnt signaling components, including Frizzled 2 (Fz2), a Wnt receptor, and Wnt4. Most Frizzled-mediated Wnt signaling is coupled to the canonical β-catenin signaling pathway, which includes the activation of Disheveled, inhibition of GSK3, nuclear accumulation of β-catenin and activation of Wnt target genes (Clevers and Nusse 2012). Frizzled also function in the planar cell polarity pathway and the Wnt/calcium pathway (Gao and Chen 2010).

The remaining three candidates possess disparate cellular functions, and we therefore grouped them into an “other” group. COP9 signalosome subunit5 (CSN5), a subunit5 of the COP9 signalosome, is an isopeptidase that deNEDDylates the cullin subunit of E3-cullin RING ubiquitin ligases (Adler *et al.*, 2008). This modification leads to decreased ubiquitin ligase activity, reducing the efficiency of the ubiquitin conjugation pathway (von Arnim 2003). Rasp is an acyltransferase enzyme that adds essential N-terminal palmitate modifications to the secreted signaling domains of Hedgehog (Hh) and Spitz (Micchelli *et al.*, 2002; Miura *et al.*, 2006). Spitz modification by Rasp restricts Spitz diffusion to increase the local concentration of the protein, while N-terminal palmitoylation of Hh is required for its activity in regulating embryonic and larval patterning. Inner centromere protein (Incenp) is a scaffold protein of the Chromosomal Passenger Complex that controls mitosis and meiosis including kinetochore-microtubule attachment, spindle assembly, and cytokinesis (van der Horst and Lens 2014). Aside from Tra, Sxl, Rasp and Abd-A, all of the other 16 candidates have human orthologs, implying a conserved role for many of the candidates in ovary morphogenesis.

### Eight candidates do not control formation of the GSC niche

**Figure 4:**
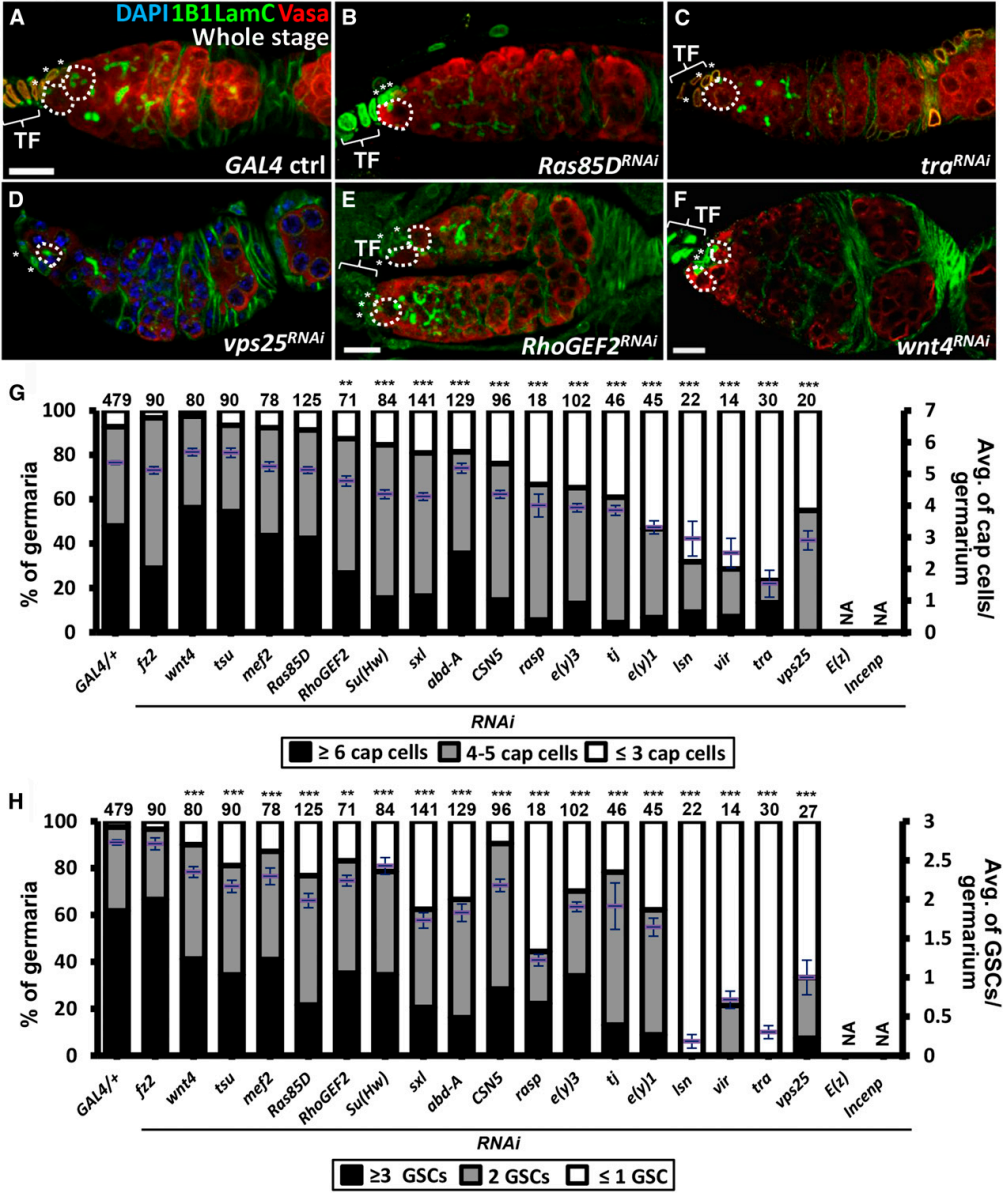
Candidates function in the soma during development to control GSC and niche cap cell numbers. (A-F) One-day-old *GAL4* control (A), *Ras85D^RNAi^* (B), *tra^RNAi^* (C), *vps25^RNAi^* (D), *RhoGEF2^RNAi^* (E), and *wnt4^RNAi^* (F) knockdown germaria, stained for LamC (green, terminal filament and cap cell nuclear envelopes), 1B1 (green, fusomes), Vasa (red, germ cells) and DAPI in D (blue, DNA). (G and H) Cap cell (G) and GSC numbers (H) in newly eclosed females were counted. Number of germaria analyzed are shown above each bar. Error bar, SEM. **P < 0.001, ***P < 0.001. Dashed circles outline GSCs. Asterisks indicate cap cells. Baskets indicate terminal filament (TF). Scale bar, 10 μm. *RNAi* was expressed throughout developmental stages (whole stage) at 29 °C. The genotype of the *GAL4* control is *bab1-GAL4/+*.

To test whether any of the 20 candidates are involved in niche and GSC formation, we knocked down each of them throughout development by *bab1-GAL4* and examined the number of niche cap cells and GSCs in 1-day-old germaria (Figure 4 and Table S2). At this stage, cap cells were clearly recognizable by their anterior location in the germarium, LamC nuclear envelope staining and rounded shape, while GSCs were identified by the anterior position of their fusome (recognized by 1B1 staining), which abuts cap cells (Xie and Spradling 2000). Because cap cell and GSC numbers vary from germarium to germarium, we analyzed both the proportion of germaria carrying a certain number of GSCs and cap cells, as well as analyzing the average numbers of GSCs and cap cells (for statistical analysis). For example, in *GAL4* controls (*bab1-GAL4/+*), approximately 90% of germaria carried more than four cap cells (average number of cap cells per germarium is 5.4 ± 1.3, n = 381 germaria) and most germaria contained more than two GSCs (average number of GSCs per germarium is 2.8 ± 0.7, n = 385 germaria) (Figure 4A, G and H). After knockdown of *fz2*, *wnt4*, *tsu*, *mef2*, *Ras85D*, *RhoGEF2*, *Su(Hw)* or *CSN5* in the soma, approximately more 80% of germaria still contained at least four cap cells and two GSCs in each niche, suggesting that these genes are not critical for niche formation.

### Seven candidates with novel function to control formation of the GSC niche

Knocking down the rest of the genes resulted in no more than 60% of germaria containing at least four cap cells and two GSCs; these candidates were selected for the second screen. For example, although 80% of *sxl*- and *abd-A-KD* germaria contained at least four cap cells (an average of 4.5 niche cap cells), only 60% of germaria carried two or more GSCs (an average of 1.7 GSCs), suggesting that niche function may be disturbed. Knockdown of *rasp*, *e(y)3*, *tj*, *e(y)1*, *lsn*, *vir*, *vps25*, and *tra* caused dramatic reductions of both niche cap cell and GSC numbers. Niche cap cells and GSCs were not counted in *E(z)* and *incenp-KD* germaria because of confounding malformations in the germaria. These results raise two possibilities. First, Rasp, E(y)3, Tj, E(y)1, Lsn, Vir, Tra, Vps25, E(z) and Incenp may participate in general ovary development, thus affecting niche formation. Second, the genes may have specific functions that only regulate certain aspects of ovary development, including niche formation.

**Figure 5:**
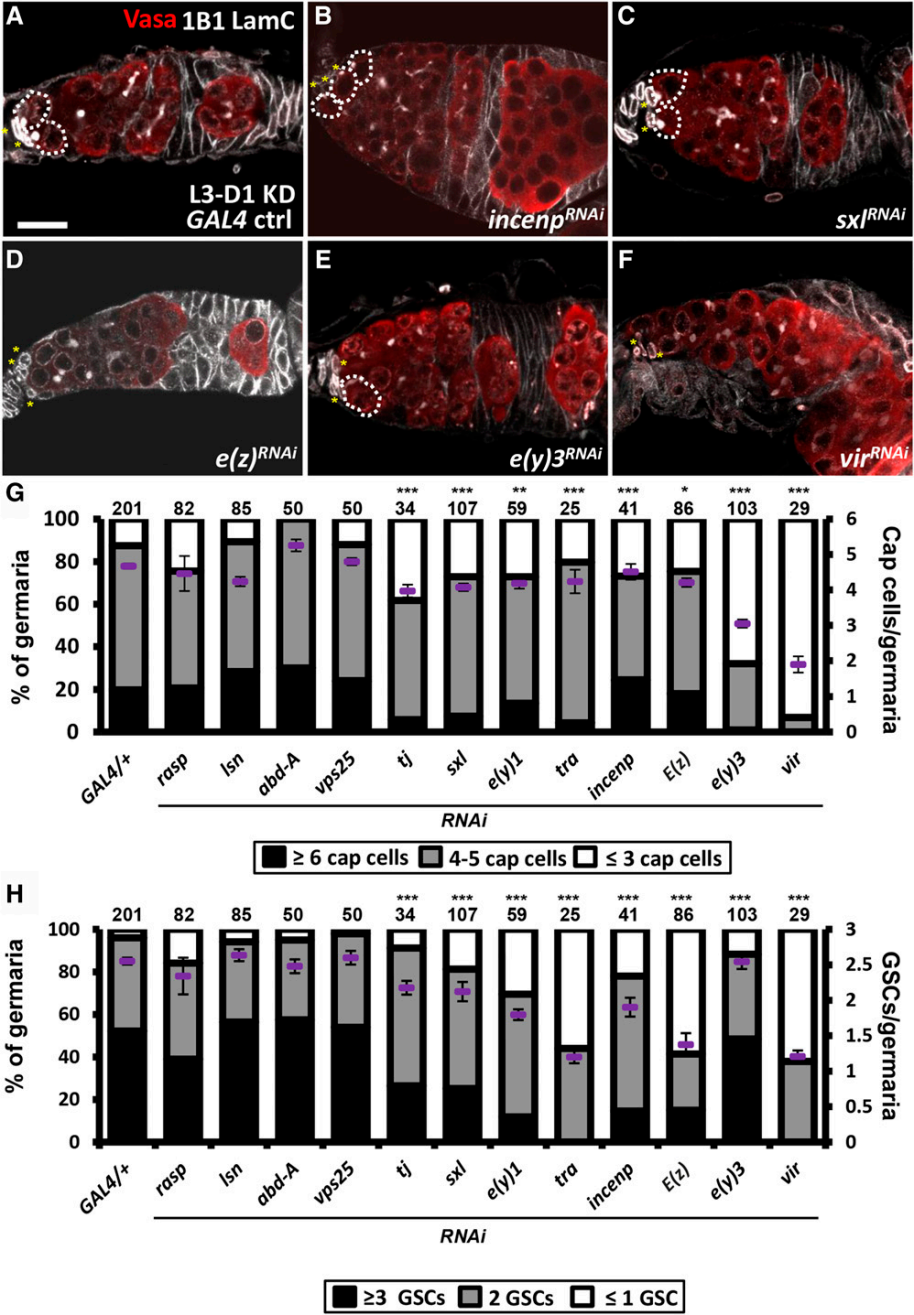
Candidates function in the soma to control GSC and niche cap cell number during larval-pupal transition. (A-F) One-day-old *GAL4* control (A), *incenp^RNAi^* (B), *sxl^RNAi^* (C), *E(z)^RNAi^* (D), *e(y)3^RNAi^* (E), and *vir^RNAi^* (F) knockdown germaria stained for LamC (gray, terminal filament and cap cell nuclear envelopes), 1B1 (gray, fusomes), and Vasa (red, germ cells) and DAPI in D (blue, DNA). (G and H) Cap cell (G) and GSC numbers (H) in newly eclosed females. Number of germaria analyzed are shown above each bar. Error bar, SEM. *P < 0.05, **P < 0.01, ***P < 0.001. Dashed circles outline GSCs. Asterisks indicate cap cells. Scale bar, 10 μm. *RNAi* was expressed from L3 to Day 1 (L3-D1 KD) at 29 °C. The genotype of the *GAL4* control is *bab1-GAL4/*+.

The Larval-pupal transition is the critical stage for niche cap cell formation and GSC recruitment (Song *et al.*, 2007). To select candidates that are involved in these processes, we used *bab1-GAL4* under the control of GAL80^ts^ to knockdown *abd-A*, *sxl*, *rasp*, *e(y)3*, *tj, e(y)1*, *lsn*, *vir*, *vps25*, *incenp*, *E(z)* and *tra* from the third-instar larvae (L3) stage to the adult stage and then counted niche cap cell and GSC numbers (Figure 5 and Table S2). Compared to controls (*bab1-GAL4/+*), *rasp*, *abd-A*-, *lsn*- and *vps25-KD* germaria did not show reductions in either niche cap cell or GSC numbers. Thus, we conclude that the homeobox-containing transcription factor, Abd-A, and the components of the ESCRT-II complex, Lsn and Vps25, do not control GSC-niche unit formation. Instead, vesicle trafficking, mediated by ESCRT-II in the soma, is expected to play a key role in ovary development prior to the L3 stage. Diminished expression of the other candidates resulted in significant reductions of both GSC and niche cap cell numbers, suggesting that these genes play direct roles in establishing the GSC-niche unit. Notably, Tj was reported to specify niche cap cell fate (Lai *et al.*, 2017), validating our screen results. The results were further confirmed using independent *RNAi* lines (Figure S2), which exhibited even stronger phenotypes.

In summary, we have identified 20 genes that function in the soma to guide normal ovary morphogenesis and reproduction. Among these genes, *Tj* and seven others (*sxl*, *tra*, *vir*, *e(z)*, *e(y)1*, *e(y)3* and *incenp*) are potentially involved in niche formation and GSC recruitment. Sxl, Tra, and Vir belong to the feminizing pathway, in which Vir regulates female-specific Sxl splicing, and then the female isoform of Sxl mediates female-specific splicing of Tra to switch on the female pathway (Penalva and Sánchez 2003). It has been shown that the sexual identity of female germ cells is reversed in the presence of male somatic cells (Casper and Van Doren 2006). In our results, knockdown of *sxl*, *tra* or *vir* causes a reduction of niche cap cell number, suggesting that sex determining genes may promote the formation of a female GSC niche to house female GSCs. E(z), a component of PRC2, is a histone transferase that trimethylates histone H3 at lysine 27 (Lund and van Lohuizen 2004). In male germ cells, it has been reported that E(z) controls dedifferentiation during aging to maintain the GSC pool (Eun *et al.*, 2017). Interestingly, E(z) in male somatic gonadal cells controls germ cell identity, and as such, knockdown of *e(z)* in the soma leads to transformation of germ cells into somatic cells (Eun *et al.*, 2014). However, the role of E(z) in female ovarian somatic cells or the GSC niche has not been explored. Similarly, the roles of E(y)1 (a component of the Transcription factor II D complex) and Incenp (a component of the chromosome passenger complex) in the formation of the GSC niche and GSC recruitment are not known. Further investigations will be required to understand whether these candidates contribute to niche formation by affecting cell fate determination, cell survival, cell division, migration or adhesion. Our results have uncovered possible candidate signaling pathways that may participate in the establishment of GSC niche in *Drosophila*, and it is likely that these signaling events are conserved in other species via expression of orthologous genes.
